# Supplementary material for: Meiosis-specific distal cohesion site decoupled from the kinetochore
Source: Nat Commun. 2025 Mar 3;16:2116. doi: 10.1038/s41467-025-57438-w (PMC11876576; doi:10.1038/s41467-025-57438-w)
Supplement: Supplementary file 1 — Supplementary Information [file 41467_2025_57438_MOESM1_ESM.pdf]

Supplementary Figures

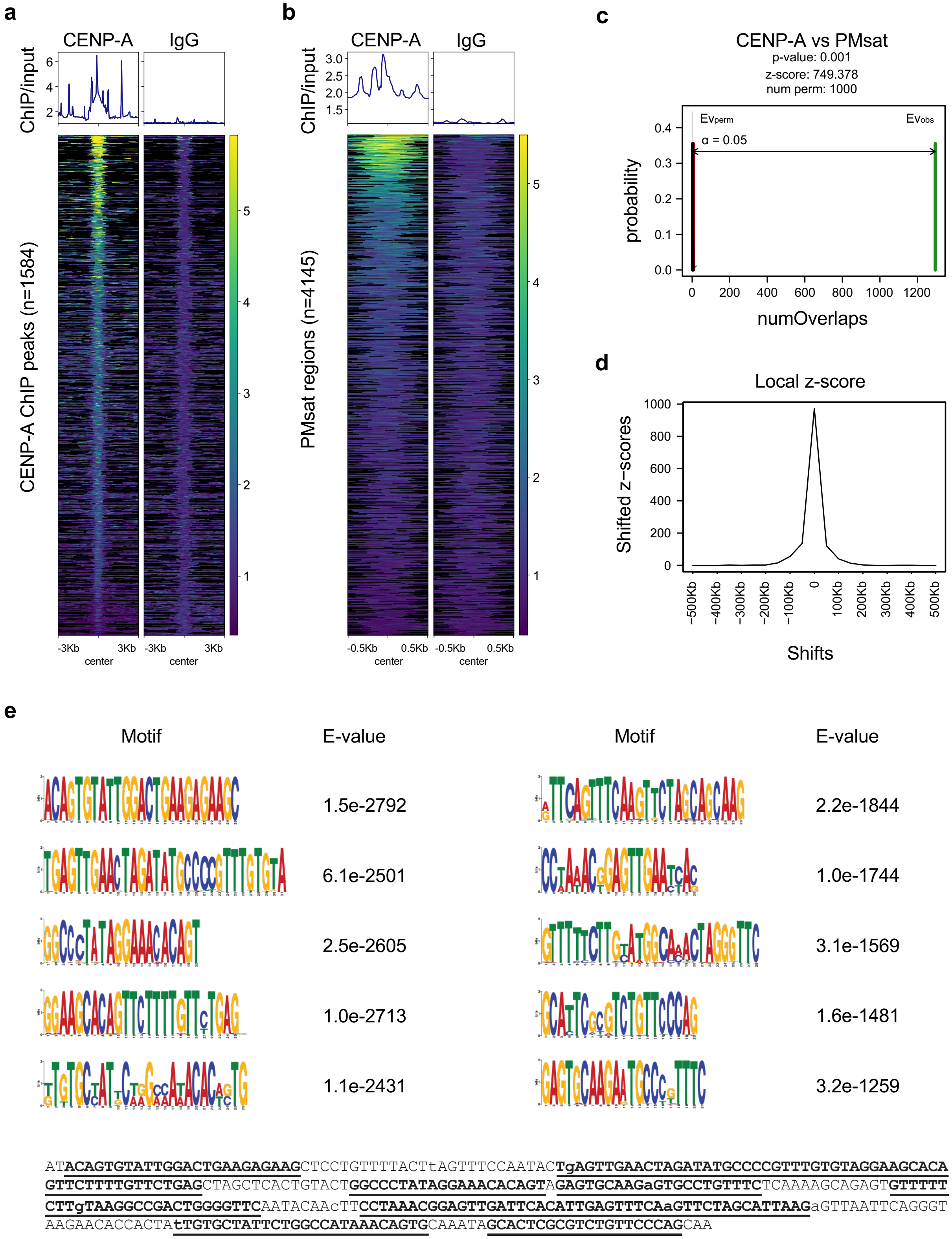

Supplementary Figure 1. PMsat is the major centromeric DNA in *Peromyscus maniculatus*. a, Profile plot and heatmap of CENP-A and IgG signal (ratio over input) at CENP-A peaks. **b,** Profile plot and heatmap of CENP-A and IgG signal (ratio over input) at PMsat regions. **c,** Representation of the permutation test results to assess association between CENP-A enriched regions and PMsat regions. The number of overlaps was used as evaluation function. The association is highly significant, as the observed value (EV_{obs}) is very distant from the mean of number of overlaps with randomized regions (EV_{perm}) and from the limit of significance of the random distribution (red line). **d,** Local Z-score plot showing that the association between CENP-A and PMsat regions is highly dependent on their exact position. **e,** Top 10 de novo identified motifs enriched at CENP-A peaks. The motifs all overlap with portions of the PMsat consensus sequence shown at the bottom. The portions represented by the motifs are highlighted by underlines. Source data are provided as a Source Data file.

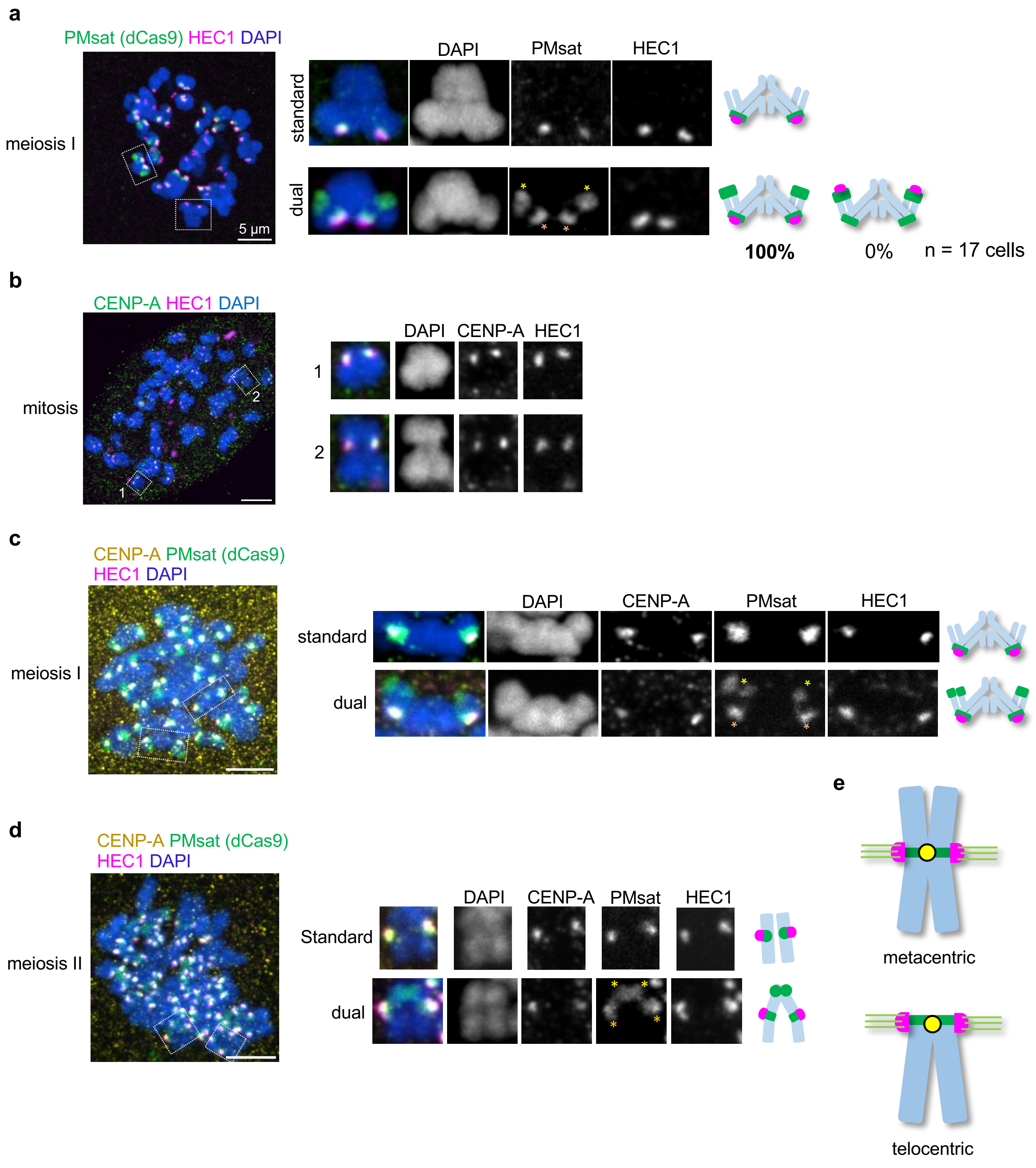

Supplementary Figure 2. Kinetochores assemble at internal PMsat in both mitosis and meiosis in *Peromyscus maniculatus*. **a**, *P. maniculatus* meiosis I oocytes expressing dCas9-EGFP and gRNA targeting PMsat were fixed and stained for HEC1. The proportion of chromosomes that assemble kinetochores at internal PMsat and telomeric PMsat was quantified; n = 17 cells from three independent experiments were examined. **b-d**, *P. maniculatus* mitotic cells (b) and meiosis I (c) and meiosis II (d) oocytes expressing dCas9-mCherry with gRNA targeting PMsat were fixed and stained for CENP-A and HEC1. n = 11, 12, and 15 cells from three independent experiments were analyzed for mitosis, meiosis I, and meiosis II, respectively. The images are maximum projections showing all the chromosomes (left) and optical sections to show individual chromosomes (right); asterisks denote the chromosomal location of internal PMsat (orange) and telomeric PMsat (yellow) on dual PMsat chromosomes; scale bars, 5 µm. **e**, Schematic illustrating metacentric and telocentric chromosomes; DNA, grey; centromere DNA, green; kinetochores, magenta; cohesin, yellow; spindle microtubules, light green.

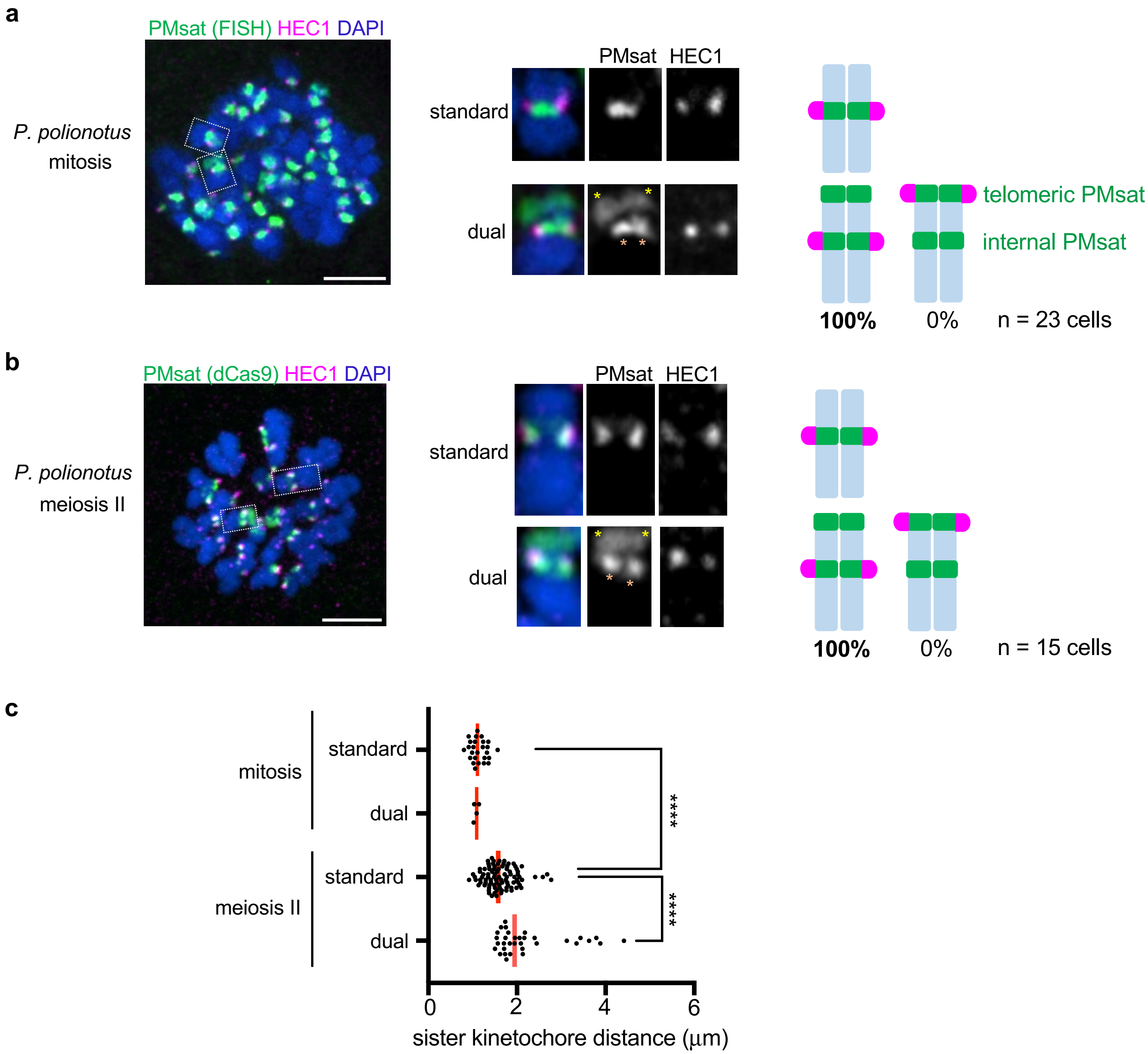

Supplementary Figure 3. Kinetochores assemble at internal PMsat also in *Peromyscus polionotus*. a,**b**, *P. polionotus* mitotic cells (a) and meiosis II oocytes expressing dCas9-EGFP with gRNA targeting PMsat (b) were fixed and stained for HEC1. For the mitotic cells, PMsat was labeled by Oligopaint. The proportion of chromosomes that assemble kinetochores at internal PMsat and telomeric PMsat was quantified for mitosis and meiosis II; n = 23 and 15 cells from three independent experiments were analyzed for mitosis (a) and meiosis II (b), respectively. The images are maximum projections showing all the chromosomes (left) and optical sections to show individual chromosomes (right); asterisks denote the chromosomal location of internal PMsat (orange) and telomeric PMsat (yellow) on dual PMsat chromosomes; scale bars, 5 µm. **c**, Quantification of sister-kinetochore distances in mitosis and meiosis II using HEC1 as a kinetochore marker; note that images acquired for Fig. 2a were used for this analysis; n = 13 and 46 cells from three and 11 independent experiments were analyzed for mitosis and meiosis II, respectively; unpaired two-tailed Mann- Whitney test was used to analyze statistical significance; $****P < 0.0001$. Source data are provided as a Source Data file.

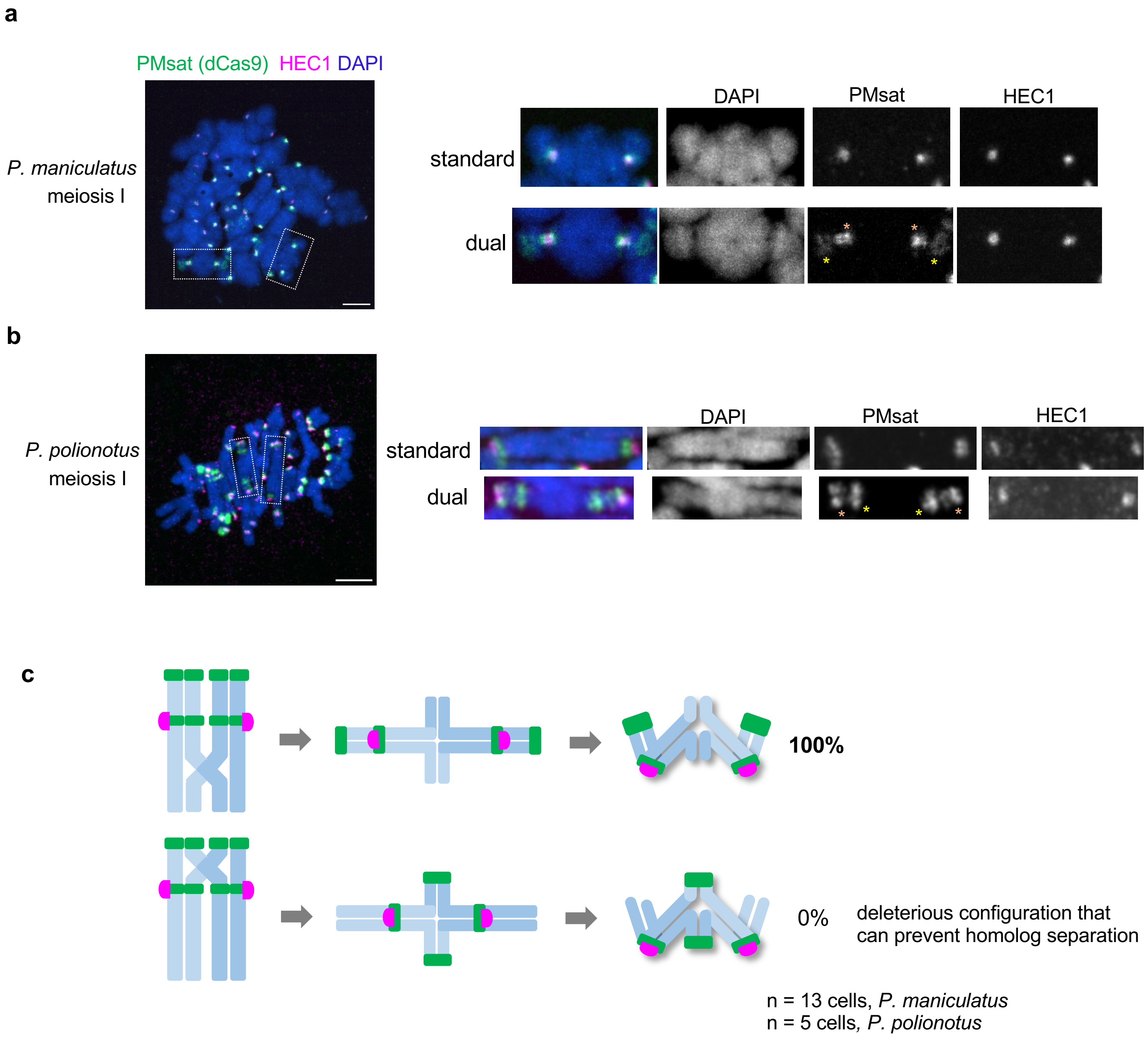

Supplementary Figure 4. Crossover formation appears to be suppressed between telomeric and internal PMsat. a-c, *P. maniculatus* meiosis I oocytes expressing dCas9-mCherry (a) and *P. polionotus* meiosis I oocytes expressing dCas9-EGFP (b) together with gRNA targeting PMsat were fixed and stained for HEC1, and the crossover pattern was quantified (c); n = 13 and 5 cells were analyzed from five and two independent experiments for *P. maniculatus* (a) and *P. polionotus* (b), respectively. The images are maximum projections showing all the chromosomes (left) and optical sections to show individual chromosomes (right); asterisks denote the chromosomal location of internal PMsat (orange) and telomeric PMsat (yellow) on dual PMsat chromosomes; scale bars, 5 µm.

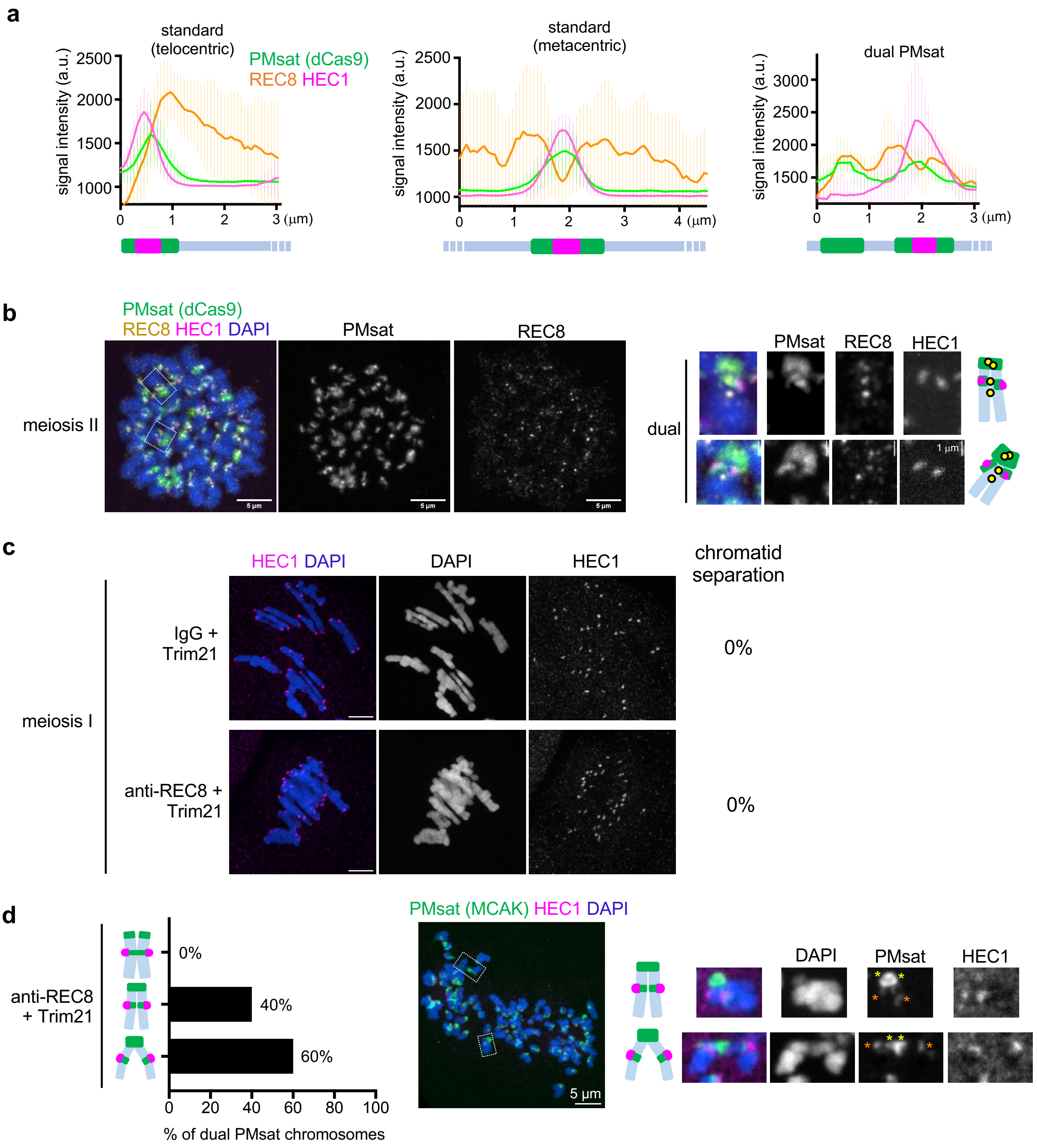

Supplementary Figure 5. Telomeric PMsat coheres sister chromatids in meiosis II by REC8 cohesin. a, Line scans of the signal intensities of REC8, HEC1, and PMsat along the meiosis I chromosome were performed using the images from Fig. 2b; n = 14 cells from three independent experiments were analyzed; lines indicate the mean values and error bars represents SD. REC8 showed an enrichment around the PMsat loci in meiosis I oocytes except for the kinetochore region. **b**, Additional examples of REC8 staining in *P. maniculatus* meiosis II oocytes from the experiment in Fig. 2b. **c**, *P. maniculatus* meiosis I oocytes microinjected with mCherry-Trim21 mRNA together with either control IgG antibody or anti-REC8 antibody were fixed at metaphase I and stained for HEC1. Microinjecting mCherry-Trim21 mRNA with the anti-REC8 antibody in meiosis I oocytes did not cause chromosome separation in metaphase I, likely due to the lower expression of mCherry-Trim21 at this stage. This observation confirms that the sister-chromatid separation (Fig. 2c) indeed occurred in meiosis II rather than in meiosis I; n = 5 and 8 cells from two independent experiments for the IgG and anti-REC8 antibody, respectively. **d**, Unseparated dual PMsat chromosomes in the REC8 Trim-Away experiment in Fig. 2c were categorized into three groups based on their cohesion pattern; n = 15 cells from three independent experiments were analyzed. Majority of unseparated dual PMsat chromosomes were cohered at telomeric PMsat, supporting the idea that telomeric PMsat is the major cohesion site of the chromosome. Asterisks denote the chromosomal location of internal PMsat (orange) and telomeric PMsat (yellow) on dual PMsat chromosomes. Source data are provided as a Source Data file.

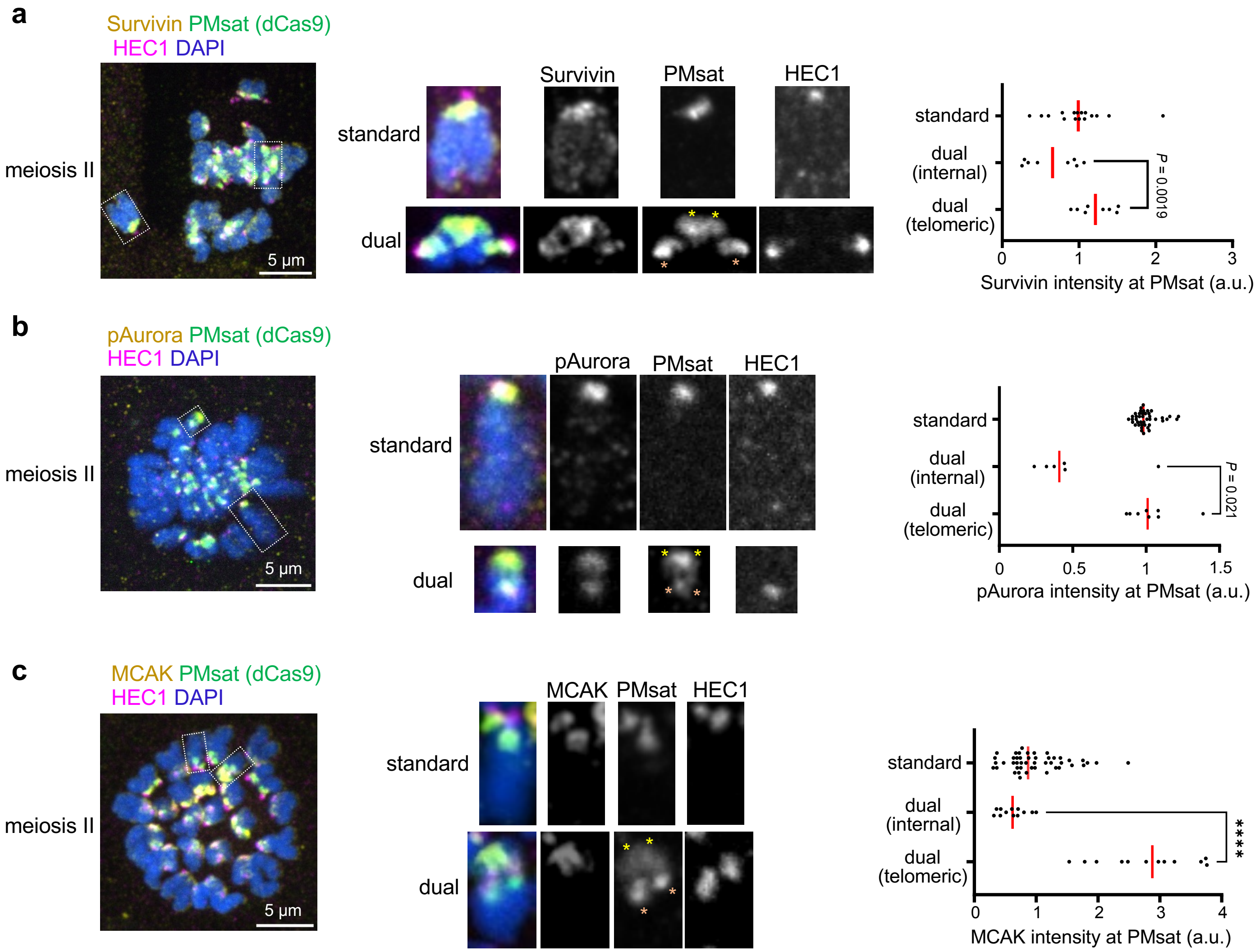

Supplementary Figure 6. Telomeric PMsat assembles a pericentromere-like structure in *Peromyscus maniculatus* meiosis II oocytes. a-c, *P. maniculatus* meiosis II oocytes expressing dCas9-EGFP (a, c) or dCas9-mCherry (b) with gRNA targeting PMsat were fixed and stained for HEC1 together with Survivin (a), pAurora (b), and MCAK (c). Signal intensities of Survivin, pAurora, and MCAK at PMsat were quantified; each dot represents one chromosome; n = 14, 16, and 10 oocytes from three independent experiments were analyzed for Survivin, pAurora, and MCAK, respectively; unpaired two-sided Mann-Whitney test was used to analyze statistical significance, exact *P* values are in the graph except for ****$P < 0.0001$; red line, median. The images are maximum projections showing all the chromosomes (left) and optical sections to show individual chromosomes (right); asterisks denote the chromosomal location of internal PMsat (orange) and telomeric PMsat (yellow) on dual PMsat chromosomes; scale bars, 5 μm. Source data are provided as a Source Data file.

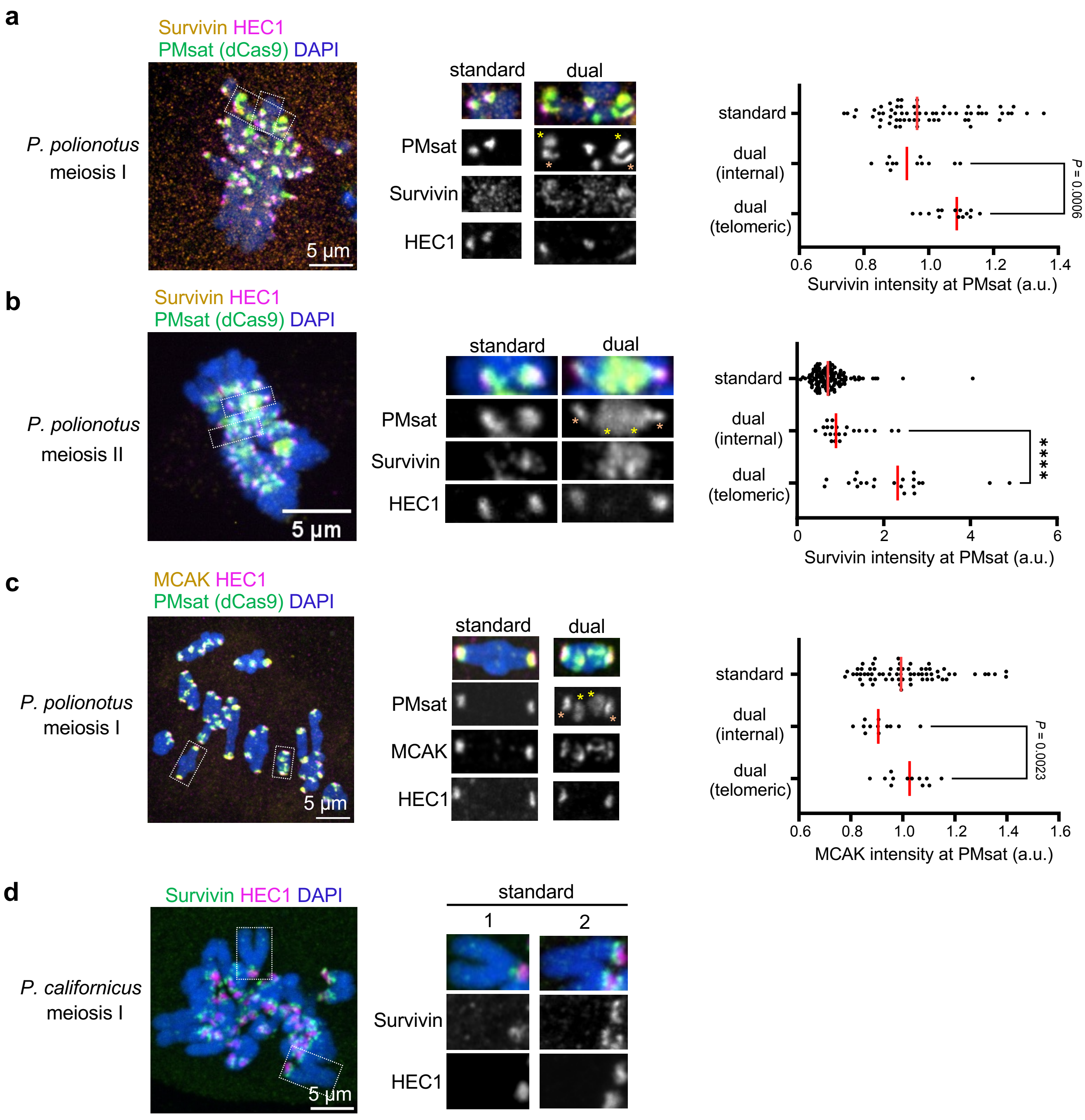

Supplementary Figure 7. Telomeric PMsat acts as an ectopic pericentromere-like structure also in *Peromyscus polionotus* oocytes. a-c, *P. polionotus* meiosis I (a,c) and meiosis II (b) oocytes expressing dCas9-EGFP with gRNA targeting PMsat were fixed and stained for HEC1 together with Survivin (a,b), or MCAK (c). Signal intensities of Survivin and MCAK at PMsat were quantified; each dot represents one chromosome; n = 98, 156, and 91 chromosomes from three independent experiments were analyzed in a, b, and c, respectively; unpaired two-tailed Mann-Whitney test was used to analyze statistical significance; exact P values are in the graphs except for $^{****}P < 0.0001$; red line, median. **d**,*Peromyscus californicus* meiosis I oocytes were fixed and stained for Survivin and HEC1. Two representative chromosomes are shown on the right, indicating the co-localization of Survivin and the kinetochore (HEC1); n = 26 cells from two independent experiments were analyzed. The images are maximum projections showing all the chromosomes (left) and optical sections to show individual chromosomes (right); asterisks denote the chromosomal location of internal PMsat (orange) and telomeric PMsat (yellow) on dual PMsat chromosomes; scale bars, 5 µm. Source data are provided as a Source Data file.

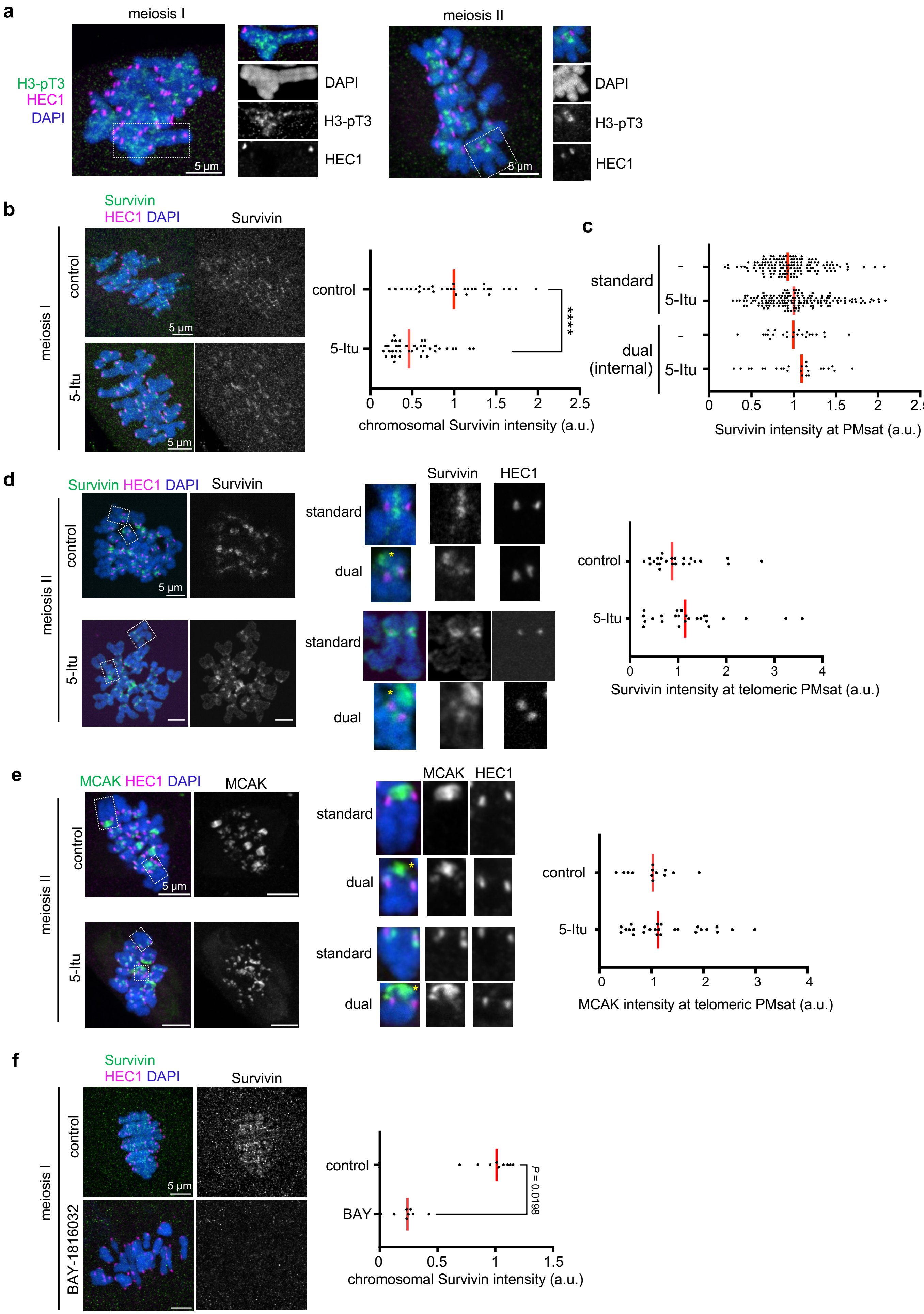

a
meiosis I
meiosis II
H3-pT3
HEC1
DAPI
DAPI
H3-pT3
HEC1
5 µm
b
Survivin
HEC1 DAPI
Survivin
meiosis I
control
5-Itu
5 µm
control
5-Itu
****
0
0.5
1
1.5
2
2.5
chromosomal Survivin intensity (a.u.)
c
standard
dual (internal)
-
5-Itu
Survivin intensity at PMsat (a.u.)
d
Survivin HEC1 DAPI
Survivin
HEC1
meiosis II
standard
dual
Survivin intensity at telomeric PMsat (a.u.)
e
MCAK HEC1 DAPI
MCAK
MCAK intensity at telomeric PMsat (a.u.)
f
BAY-1816032
BAY
P = 0.0198

Supplementary Figure 8. Inhibiting Haspin and BUB1 kinases in *Peromyscus* oocytes. **a**, *P. maniculatus* meiosis I and II oocytes were fixed and stained for H3-pT3 and HEC1; n = 16 cells were analyzed from three independent experiments. **b**, *P. maniculatus* meiosis I oocytes treated with 0.5 µM 5-Itu were fixed at metaphase I and stained for Survivin and HEC1. Signal intensities of Survivin on the chromosome were quantified; each dot represents one chromosome, n = 31 and 36 cells from three independent experiments for control and the 5-Itu-treated group, respectively; unpaired two-sided Mann-Whitney test was used to analyze statistical significance; red line, median. **c**, Survivin signals at PMsat were quantified using the images acquired for Fig. 5c; n = 26 and 23 cells from three independent experiments for control and the 5-Itu-treated group, respectively; red line, median. **d**, *P. maniculatus* meiosis I oocytes treated with 5-iodotubercidin (5-Itu) were matured to meiosis II, fixed, and stained for HEC1 and Survivin. Signal intensities of Survivin at telomeric PMsat were quantified; each dot represents one chromosome, n = 26 and 23 chromosomes from three independent experiments for control and the 5-Itu-treated group, respectively; red line, median. **e**, *P. maniculatus* meiosis I oocytes treated with 0.5 µM 5-Itu were matured to meiosis II, fixed, and stained for MCAK and HEC1. MCAK signal intensities at telomeric PMsat (each dot represents one chromosome) were quantified; n = 25 and 34 chromosomes from three independent experiments for control and the 5-Itu-treated group, respectively; red line, median. **f**, *P. maniculatus* meiosis I oocytes treated with a BUB1 inhibitor, BAY-1816032, were fixed and stained for Survivin and HEC1. Survivin signal intensities on the chromosome were quantified; n = 13 and 14 cells from four independent experiments were analyzed for control and the BAY-1816032-treated group, respectively; unpaired two-sided Mann-Whitney test was used to analyze statistical significance; red line, median. Exact *P* values are in the graph except for $^{****}P < 0.0001$. The images are maximum projections showing all the chromosomes (left) and optical sections to show individual chromosomes (right); asterisks denote the chromosomal location of internal PMsat (orange) and telomeric PMsat (yellow) on dual PMsat chromosomes; scale bars, 5 µm. Source data are provided as a Source Data file.

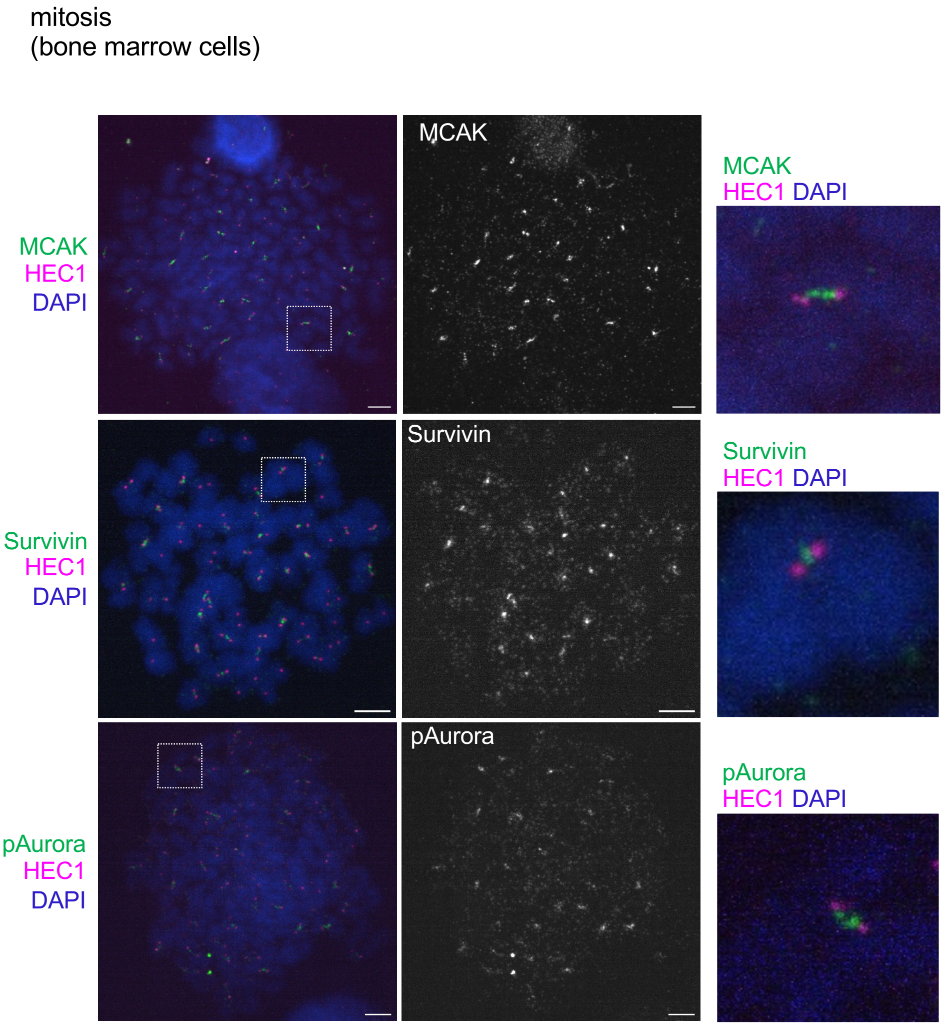

Supplementary Figure 9. No ectopic pericentromere-like structure formation in bone marrow mitotic cells. *P. maniculatus* bone marrow cells were fixed and stained for HEC1 together with MCAK (top), Survivin (middle), and pAurora (bottom). MCAK, Survivin, and pAurora consistently localized between sister kinetochores labeled by HEC1, and we did not find any mitotic cells that formed ectopic pericentromere-like structures; n = 7, 9, and 9 cells were analyzed from three independent experiments for MCAK, Survivin, and pAurora, respectively. The images are maximum projections showing all the chromosomes (left) and optical sections to show individual chromosomes (right); scale bars, 5 μm.

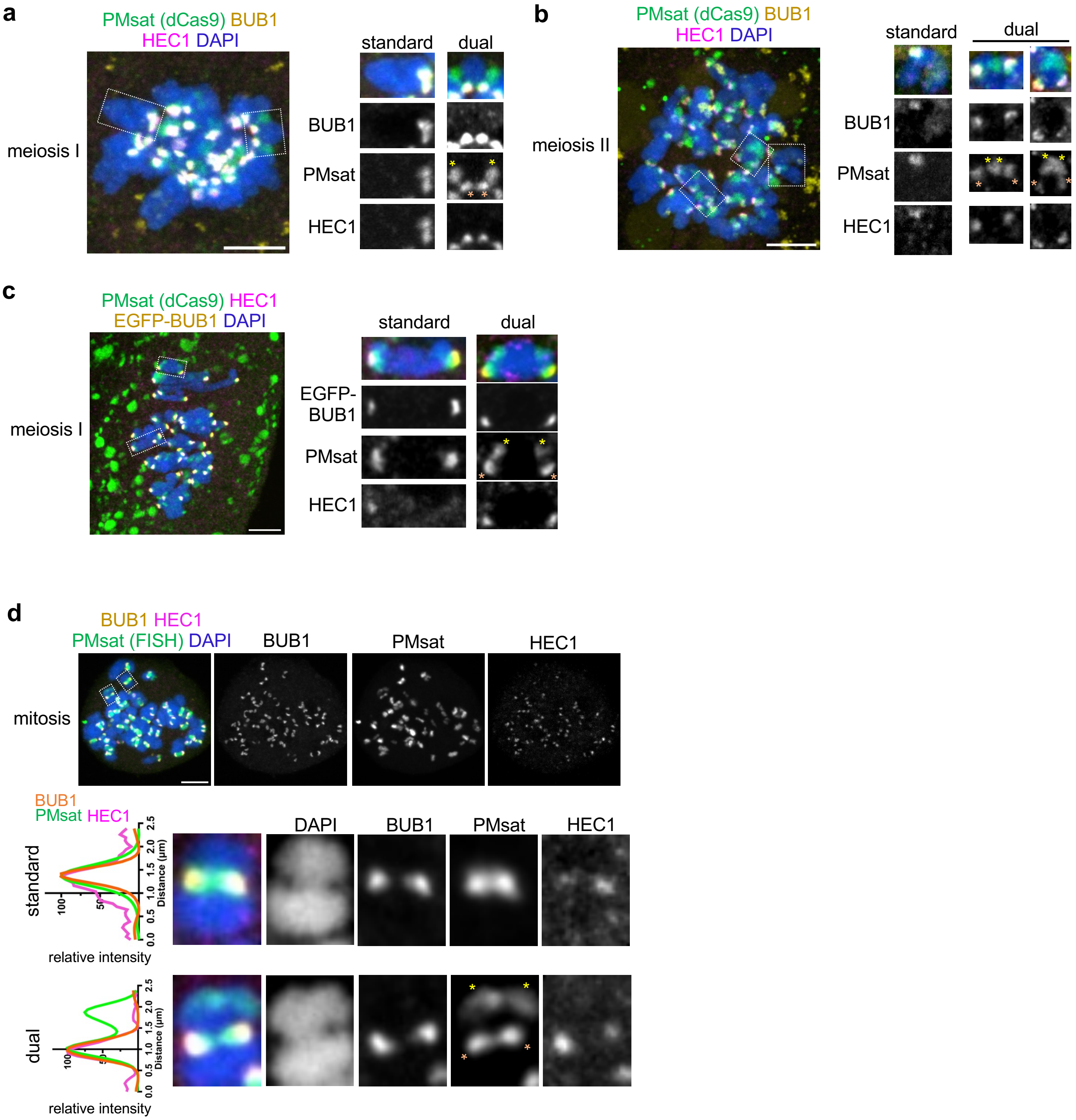

Supplementary Figure 10. No obvious BUB1 enrichment at telomeric PMsat. a,**b**, *P. maniculatus* meiosis I (a) and meiosis II (b) oocytes expressing dCas9-mCherry with gRNA targeting PMsat were fixed and stained for HEC1 and BUB1. In both meiosis I and II, no obvious enrichment of BUB1 was observed at telomeric PMsat; n = 8 and 27 cells from three independent experiments were analyzed for meiosis I and II, respectively. **c**, *P. maniculatus* meiosis I oocytes expressing EGFP-BUB1 and dCas9-mCherry with gRNA targeting PMsat were fixed and stained for HEC1. Overexpressed EGFP-BUB1 did not localize to telomeric PMsat; n = 21 cells from three independent experiments. **d**, *P. maniculatus* granulosa cells arrested in mitosis by Nocodazole were fixed and stained for HEC1 and BUB1. Immunostained cells were then labeled for PMsat using the Oligopaint technique. n = 10 cells from three independent experiments were analyzed. Line scans of the signal intensities of BUB1, PMsat, and HEC1 were performed along the chromosome. BUB1 exclusively enriched at internal PMsat in mitotic cells. The images are maximum projections showing all the chromosomes (left for a-c, top for d) and optical sections to show individual chromosomes (right for a-c, bottom for d); asterisks denote the chromosomal location of internal PMsat (orange) and telomeric PMsat (yellow) on dual PMsat chromosomes; scale bars, 5 µm. Source data are provided as a Source Data file.

Supplementary Table 1

Main Figures

		Experiment repeats	Number of chromosomes or centromeres	Number of Cells
Fig 1c	mitosis	3		24
Fig 1d	meiosis II	5		56
Fig 2a	mitosis	3	52	13
	meiosis II	11	38	46
Fig 2b	meiosis I	3		14
	meiosis II	3		9
Fig 2c	IgG	3		23
	anti-REC8			15
Fig 3a		3	152	
Fig 3b	Control	4		26
	OA			33
Fig 4b		3	32	
Fig 4c		3	210	
Fig 4d		3	87	
Fig 5b	Control	3		16
	5-Itu			15
Fig 5c	Control	3	24	
	5-Itu		25	
Fig 5d	Control	3	28	13
	BAY		42	11
Fig 5e	Control	3	16	16
	BAY		7	10
Fig 5f	Control	4	122	
	BAY		141	
Fig 6a		3	197	13
Fig 6b		3	147	21
Fig 6c		3	128	11

Supplementary Figures

		Experiment repeats	Number of chromosomes or centromeres	Number of Cells
Fig 2a		3		17
Fig 2b		3		11
Fig 2c		3		12
Fig 2d		3		15
Fig 3a		3		23
Fig 3b		3		15
Fig. 3c		same with main Fig. 2a		
Fig 4a		5		13
Fig 4b		3		8
Fig. 5a		same with main Fig. 2b		
Fig 5b		additional example from the experiment in Fig. 2b		
Fig 5c	IgG	2		5
	anti-REC8			8
Fig 5d		same with main Fig. 2c		
Fig 6a		3	32	14
Fig 6b		3	56	16
Fig 6c		3	70	10
Fig 7a		3	90	
Fig 7b		3	170	
Fig 7c		3	90	
Fig 7d		3		30
Fig 8a		3		16
Fig 8b	Control	3		32
	5-Itu			39
Fig. 8c		same with main Fig. 5c		
Fig 8d	Control	3	24	
	5-Itu		25	
Fig 8e	Control	3	13	
	5-ITu		26	
Fig 8f	Control	4		9
	BAY			8
Fig 9	MCAK	3		7
	Survivin			9
	pAurora			9
Fig 10a		3		8
Fig 10b		3		27
Fig 10c		3		21
Fig 10d		3		10
