# Supplementary material for: Meiosis-specific distal cohesion site decoupled from the kinetochore
Source: Nat Commun. 2025 Mar 3;16:2116. doi: 10.1038/s41467-025-57438-w (PMC11876576; doi:10.1038/s41467-025-57438-w)
Supplement: Supplementary file 2 — Reporting Summary [file 41467_2025_57438_MOESM2_ESM.pdf]

nature portfolio

Corresponding author(s): Takashi Akera

Last updated by author(s): Jan 30, 2025

Reporting Summary

Nature Portfolio wishes to improve the reproducibility of the work that we publish. This form provides structure for consistency and transparency in reporting. For further information on Nature Portfolio policies, see our Editorial Policies and the Editorial Policy Checklist.

Statistics

For all statistical analyses, confirm that the following items are present in the figure legend, table legend, main text, or Methods section.

n/a	Confirmed	
☐	☒	The exact sample size (n) for each experimental group/condition, given as a discrete number and unit of measurement
☐	☒	A statement on whether measurements were taken from distinct samples or whether the same sample was measured repeatedly
☐	☒	The statistical test(s) used AND whether they are one- or two-sided<br>*Only common tests should be described solely by name; describe more complex techniques in the Methods section.*
☒	☐	A description of all covariates tested
☒	☐	A description of any assumptions or corrections, such as tests of normality and adjustment for multiple comparisons
☐	☒	A full description of the statistical parameters including central tendency (e.g. means) or other basic estimates (e.g. regression coefficient) AND variation (e.g. standard deviation) or associated estimates of uncertainty (e.g. confidence intervals)
☐	☒	For null hypothesis testing, the test statistic (e.g. F, t, r) with confidence intervals, effect sizes, degrees of freedom and P value noted<br>*Give P values as exact values whenever suitable.*
☒	☐	For Bayesian analysis, information on the choice of priors and Markov chain Monte Carlo settings
☒	☐	For hierarchical and complex designs, identification of the appropriate level for tests and full reporting of outcomes
☒	☐	Estimates of effect sizes (e.g. Cohen's d, Pearson's r), indicating how they were calculated

Our web collection on statistics for biologists contains articles on many of the points above.

Software and code

Policy information about availability of computer code

Data collection

Cells were imaged using a Nikon Eclipse Ti microscope. The microscope was equipped with a 100x / 1.40 NA oil-immersion objective lens, a CSU-W1 spinning disk confocal scanner by Yokogawa, an ORCA Fusion Digital CMOS camera from Hamamatsu Photonics, and controlled laser lines at 405 nm, 488 nm, 561 nm, and 640 nm via NIS-Elements imaging software by Nikon. Confocal images were captured as Z-stacks at 0.3 µm intervals, and these images were presented as maximum intensity Z-projections unless specified in the figure legend. DNA libraries were sequenced as 100 bp paired-end reads on an Illumina NovaSeq 6000 system.

Data analysis

Fiji/ImageJ (NIH) was used to analyze all the images, and Microsoft Excel and GraphPad Prism 10 were used for statistical analyses. Read quality was assessed by fastQC v0.12.1. Reads were aligned to the genome assembly HU_Pman_2.1.3 (GCF_003704035.1) of Peromyscus maniculatus bairdii using the Burrows-Wheeler Alignment (BWA) tool v0.7.17 (bwa aln and bwa sampe commands, default settings). Sam files were then converted into bam files with SAMtools v1.19, while removing eventually unmapped and duplicated reads, and retaining only primary alignments (samtools view -F 0x4,0x400,0x100,0x800 -b -h file.sam > file.bam). Bam files were sorted and indexed with SAMtools and converted to bigwig normalized to 1x genome coverage (RPGC normalization) for each sample with deepTools v3.5.4a (bamCoverage --bam file.bam -o file.bw -of bigwig --binSize 10 --effectiveGenomeSize 2385634842 --normalizeUsing RPGC --extendReads 200). The effective genome size was calculated using the unique-kmers.py command of the tool khmer v2.1.1 (with -k 200). ChIP bigwigs were further normalized by the input using deepTools (bigwigCompare -b1 ChIP.bw -b2 input.bw -o CENPA_input_ratio.bw -of bigwig --operation ratio –skipZeroOverZero --binSize 10). Peaks were called using MACS2 v2.2.7.1 (macs2 callpeak -t ChIP.bam -c input.bam -f BAMPE -g 2385634842). Heatmaps and enrichment profiles were plotted using deepTools.
Sequences underlying CENP-A peaks were extracted with getfasta command from BEDTools v2.31.1. De novo motif finding was performed with Multiple Em for Motif Elicitation (MEME) tool from MEME Suite v5.5.5 (with options -mod anr -nmotifs 30 -minw 20 -maxw 50 -objfun classic -revcomp -markov_order 0).
To perform enrichment analysis of CENP-A at genomic regions presenting PMsat sequences, a blastn search was performed for the PMsat

consensus in the HU_Pman_2.1.3 reference genome assembly. Alignment regions that overlapped or that were at most 10 bp apart were merged using BEDTools merge command. Local Z-score analysis and permutation test (n = 1000) to assess the association between CENP-A enriched regions and PMsat regions were performed with regioneR v4.3.1.

For manuscripts utilizing custom algorithms or software that are central to the research but not yet described in published literature, software must be made available to editors and reviewers. We strongly encourage code deposition in a community repository (e.g. GitHub). See the Nature Portfolio guidelines for submitting code & software for further information.

Data

Policy information about availability of data

All manuscripts must include a data availability statement. This statement should provide the following information, where applicable:

- Accession codes, unique identifiers, or web links for publicly available datasets
- A description of any restrictions on data availability
- For clinical datasets or third party data, please ensure that the statement adheres to our policy

Source Data are provided with this paper. Data required to reproduce the results in the current study are available at Figshare (https://doi.org/10.25444/nhlbi.28001618). Raw data files for the DNA sequencing analysis have been deposited in the NCBI Sequence Read Archive (SRA) under project accession number SRA: PRJNA1196496.

Research involving human participants, their data, or biological material

Policy information about studies with human participants or human data. See also policy information about sex, gender (identity/presentation), and sexual orientation and race, ethnicity and racism.

Reporting on sex and gender	*Use the terms sex (biological attribute) and gender (shaped by social and cultural circumstances) carefully in order to avoid confusing both terms. Indicate if findings apply to only one sex or gender; describe whether sex and gender were considered in study design; whether sex and/or gender was determined based on self-reporting or assigned and methods used. Provide in the source data disaggregated sex and gender data, where this information has been collected, and if consent has been obtained for sharing of individual-level data; provide overall numbers in this Reporting Summary. Please state if this information has not been collected. Report sex- and gender-based analyses where performed, justify reasons for lack of sex- and gender-based analysis.*
Reporting on race, ethnicity, or other socially relevant groupings	*Please specify the socially constructed or socially relevant categorization variable(s) used in your manuscript and explain why they were used. Please note that such variables should not be used as proxies for other socially constructed/relevant variables (for example, race or ethnicity should not be used as a proxy for socioeconomic status). Provide clear definitions of the relevant terms used, how they were provided (by the participants/respondents, the researchers, or third parties), and the method(s) used to classify people into the different categories (e.g. self-report, census or administrative data, social media data, etc.) Please provide details about how you controlled for confounding variables in your analyses.*
Population characteristics	*Describe the covariate-relevant population characteristics of the human research participants (e.g. age, genotypic information, past and current diagnosis and treatment categories). If you filled out the behavioural & social sciences study design questions and have nothing to add here, write "See above."*
Recruitment	*Describe how participants were recruited. Outline any potential self-selection bias or other biases that may be present and how these are likely to impact results.*
Ethics oversight	*Identify the organization(s) that approved the study protocol.*

Note that full information on the approval of the study protocol must also be provided in the manuscript.

Field-specific reporting

Please select the one below that is the best fit for your research. If you are not sure, read the appropriate sections before making your selection.

☒ Life sciences ☐ Behavioural & social sciences ☐ Ecological, evolutionary & environmental sciences

For a reference copy of the document with all sections, see nature.com/documents/nr-reporting-summary-flat.pdf

Life sciences study design

All studies must disclose on these points even when the disclosure is negative.

Sample size	No statistical methods were used to predetermine sample size. Between 3 - 30 cells were analyzed per biological replicate, depending on the type of experiment. The sample size was determined to have sufficient statistical power.
Data exclusions	No data were excluded from the analysis.
Replication	All attempts of replication were successful. Data points were pooled from two to 11 independent experiments (biological replicates). The exact number of experiments for each experimental group is listed in Table S1.

Randomization	No randomization was performed.
Blinding	Data collections during imaging were blinded by only looking at DAPI signals when selecting cells.

Reporting for specific materials, systems and methods

We require information from authors about some types of materials, experimental systems and methods used in many studies. Here, indicate whether each material, system or method listed is relevant to your study. If you are not sure if a list item applies to your research, read the appropriate section before selecting a response.

Materials & experimental systems

n/a	Involved in the study	
☐	☒	Antibodies
☒	☐	Eukaryotic cell lines
☒	☐	Palaeontology and archaeology
☐	☒	Animals and other organisms
☒	☐	Clinical data
☒	☐	Dual use research of concern
☒	☐	Plants

Methods

n/a	Involved in the study	
☐	☒	ChIP-seq
☒	☐	Flow cytometry
☒	☐	MRI-based neuroimaging

Antibodies

Antibodies used

The following primary antibodies were used at the indicated delusions for both oocytes and somatic cells: rabbit anti-mouse REC8 (1:200, gift from Michael A. Lampson), mouse anti-human PP2A C subunit (1:100, EMD Millipore, cat# 05-421-AF488), rabbit anti-human Survivin (1:100, Cell Signaling Technology. cat# 2808), rabbit anti-human phospho-Aurora A (Thr288)/Aurora B (Thr232)/ Aurora C (Thr198), pAurora (1:100, Cell Signaling Technology, cat# 2914S), rabbit anti-human MCAK (1:1000, gift from Duane Compton), rabbit anti-histone H3-pT3 (1:100, Active Motif, cat# 39154), sheep polyclonal anti human-BUB1 antibody, SB1.3 (1:50, gift from Stephen Taylor), rabbit anti-histone H2A-pT120 (1:2000, Active motif, cat# 39391), mouse anti-human HEC1 (1:200, Santa Cruz, cat# sc-515550), CREST human autoantibody against centromere, ACA (1:100, Immunovision, cat# HCT-0100), goat anti-GFP antibody conjugated with Dylight488 (1:100, Rockland, cat# 600-141-215).
Secondary antibodies were Alexa Fluor 488–conjugated donkey anti-rabbit (1:500, Invitrogen, cat# A21206) or donkey anti-goat (1:500, Invitrogen, cat# A11057), Alexa Fluor 568–conjugated goat anti-rabbit (1:500, Invitrogen, cat# A10042), or Alexa Fluor 647–conjugated goat anti-human (1:500, Invitrogen, cat# A21445).

Validation

rabbit anti-mouse REC8 is a giift from Michael A. Lampson, and it is validated in Chiang et al., Curr Biol. 2010.
rabbit anti-human MCAK is a gift from Duane Compton, and it is validated in Illingworth et al., Development, 2010.
sheep polyclonal anti human-BUB1 antibody, SB1.3 is a gift from Stephen Taylor, and it is validated in Taylor et al JCS 2001.

The following antibodies have been validated by the suppliers.
mouse anti-human PP2A C subunit: https://www.emdmillipore.com/US/en/product/Anti-PP2A-C-subunit-Antibody-clone-1D6-Alexa-Fluor-488-Conjugate,MM_NF-05-421-AF488

rabbit anti-human Survivin: https://www.cellsignal.com/products/primary-antibodies/survivin-71g4b7-rabbit-mab/2808?srsltid=AfmBOoqXjLRHI2BK8EJLxaaCGk5xqJ-4UIdhYL1E9YAb1pJa0amlkPaO

rabbit anti-human phospho-Aurora A (Thr288)/Aurora B (Thr232)/Aurora C (Thr198): https://www.cellsignal.com/products/primary-antibodies/phospho-aurora-a-thr288-aurora-b-thr232-aurora-c-thr198-d13a11-xp-rabbit-mab/2914?srsltid=AfmBOooU7dYK_Lj3cPTMY5jOPFaRypp7RWiFTa2_QhnQfiaPSucQKn12

rabbit anti-histone H3-pT3: https://www.activemotif.com/catalog/details/39153/histone-h3-phospho-thr3-antibody-pab

rabbit anti-histone H2A-pT120: https://www.activemotif.com/catalog/details/39391/histone-h2a-phospho-thr120-antibody-pab

mouse anti-human HEC1: https://www.scbt.com/p/hec1-antibody-c-11?srsltid=AfmBOooI3Bau1Xk4MF08FIjIPhb4gahN58frmZ9k8wxFXTfdmFzneYD7

CREST human autoantibody against centromere, ACA: https://calbiotech.com/products/human-antibody-against-centromere?srsltid=AfmBOopb8NbEO7nCBTBqc9OskKdKf2McsFjtkZlhiaWsZOj-q4jQSXaY

goat anti-GFP antibody conjugated with Dylight488: https://www.rockland.com/categories/primary-antibodies/gfp-antibody-dylight-488-conjugated-600-141-215/?srsltid=AfmBOoo3Jbd3RXC1gW_JQxC9ZfQceQ3QQTGGcQCI-1Hta9QN-PVsJ2CZ

Alexa Fluor 488–conjugated donkey anti-rabbit: https://www.thermofisher.com/antibody/product/Donkey-anti-Rabbit-IgG-H-L-Highly-Cross-Adsorbed-Secondary-Antibody-Polyclonal/A-21206

Alexa Fluor 488–conjugateddonkey anti-goat: https://www.thermofisher.com/antibody/product/Donkey-anti-Goat-IgG-H-L-Cross-Adsorbed-Secondary-Antibody-Polyclonal/A-11057

Alexa Fluor 568–conjugated goat anti-rabbit: https://www.thermofisher.com/antibody/product/Donkey-anti-Rabbit-IgG-H-L-Highly-Cross-Adsorbed-Secondary-Antibody-Polyclonal/A10042

Alexa Fluor 647–conjugated goat anti-human: https://www.thermofisher.com/antibody/product/Goat-anti-Human-IgG-H-L-Cross-Adsorbed-Secondary-Antibody-Polyclonal/A-21445

Animals and other research organisms

Policy information about studies involving animals; ARRIVE guidelines recommended for reporting animal research, and Sex and Gender in Research

Laboratory animals	Peromyscus maniculatus bairdii (BW strain), Peromyscus polionotus subgriseus (PO strain) and Peromyscus californicus insignis (IS strain) mice were obtained from the Peromyscus Genetic Stock Center at the University of South Carolina (https://sc.edu/study/colleges_schools/pharmacy/centers/peromyscus_genetic_stock_center/).
Wild animals	No wild animal were used in this study.
Reporting on sex	We only used female mice, because we study female meiosis.
Field-collected samples	This study does not involve sample collections from the field.
Ethics oversight	All animal experiments were approved by the Animal Care and Use Committee (National Institutes of Health Animal Study Proposal#: H-0327) and were consistent with the National Institutes of Health guidelines.

Note that full information on the approval of the study protocol must also be provided in the manuscript.

Plants

Seed stocks	*Report on the source of all seed stocks or other plant material used. If applicable, state the seed stock centre and catalogue number. If plant specimens were collected from the field, describe the collection location, date and sampling procedures.*
Novel plant genotypes	*Describe the methods by which all novel plant genotypes were produced. This includes those generated by transgenic approaches, gene editing, chemical/radiation-based mutagenesis and hybridization. For transgenic lines, describe the transformation method, the number of independent lines analyzed and the generation upon which experiments were performed. For gene-edited lines, describe the editor used, the endogenous sequence targeted for editing, the targeting guide RNA sequence (if applicable) and how the editor was applied.*
Authentication	*Describe any authentication procedures for each seed stock used or novel genotype generated. Describe any experiments used to assess the effect of a mutation and, where applicable, how potential secondary effects (e.g. second site T-DNA insertions, mosiacism, off-target gene editing) were examined.*

ChIP-seq

Data deposition

☒ Confirm that both raw and final processed data have been deposited in a public database such as GEO.

☒ Confirm that you have deposited or provided access to graph files (e.g. BED files) for the called peaks.

Data access links<br>*May remain private before publication.*	https://dataview.ncbi.nlm.nih.gov/object/PRJNA1196496?reviewer=dpbf2khmdeu3ogd5p4c1t3b2vp
Files in database submission	chromatin input sample, control IgG ChIP seq, CENP-A ChIP seq of Peromyscus maniculatus: granulosa cells (BED files other processed files are deposited to figshare (https://doi.org/10.25444/nhlbi.28001618))
Genome browser session<br>(e.g. UCSC)	https://genome.ucsc.edu/s/pub_sessions/CENP%2DA_ChIP_peromyscus

Methodology

Replicates	One replicate
Sequencing depth	Samples were sequenced as 100bp paired-end reads, yielding 61778882x2 reads for CENP-A ChIP, 68793409x2 reads for IgG ChIP and 88407874x2 reads for input.
Antibodies	A custom guinea pig anti-CENP-A antibody was used for CENP-A ChIP; guinea pig IgG (SinoBiological, cat# CR4) was used for IgG ChIP.
Peak calling parameters	Peaks were called against the input using MACS2 v2.2.7.1 with the following command: macs2 callpeak -t ChIP.bam -c input.bam -f BAMPE -g 2385634842. The -g parameter for the effective genome size was calculated for the HU_Pman_2.1.3 (GCF_003704035.1) assembly using the tool khmer v2.1.1 (with -k 200).

Data quality	Read quality was assessed by fastQC analysis. A total of 1584 peaks (qValue <0.05) were identified for the CENP-A ChIP; 1141 peaks had fold enrichment higher than 5.
Software	The following tools were used for the ChIP-seq analysis: BWA v0.7.17 , SAMtools v1.19, deepTools v3.5.4a, MACS2 v2.2.7.1.
